# CausalBuilder: bringing the MI2CAST causal interaction annotation standard to the curator

**DOI:** 10.1093/database/baaa107

**Published:** 2021-02-24

**Authors:** Vasundra Touré, John Zobolas, Martin Kuiper, Steven Vercruysse

**Affiliations:** Department of Biology, Norwegian University of Science and Technology (NTNU), Høgskoleringen 5, 7491 Trondheim, Norway; Department of Biology, Norwegian University of Science and Technology (NTNU), Høgskoleringen 5, 7491 Trondheim, Norway; Department of Biology, Norwegian University of Science and Technology (NTNU), Høgskoleringen 5, 7491 Trondheim, Norway; Department of Biology, Norwegian University of Science and Technology (NTNU), Høgskoleringen 5, 7491 Trondheim, Norway

## Abstract

Molecular causal interactions are defined as regulatory connections between biological components. They are commonly retrieved from biological experiments and can be used for connecting biological molecules together to enable the building of regulatory computational models that represent biological systems. However, including a molecular causal interaction in a model requires assessing its relevance to that model, based on the detailed knowledge about the biomolecules, interaction type and biological context. In order to standardize the representation of this knowledge in ‘causal statements’, we recently developed the Minimum Information about a Molecular Interaction Causal Statement (MI2CAST) guidelines. Here, we introduce causalBuilder: an intuitive web-based curation interface for the annotation of molecular causal interactions that comply with the MI2CAST standard. The causalBuilder prototype essentially embeds the MI2CAST curation guidelines in its interface and makes its rules easy to follow by a curator. In addition, causalBuilder serves as an original application of the Visual Syntax Method general-purpose curation technology and provides both curators and tool developers with an interface that can be fully configured to allow focusing on selected MI2CAST concepts to annotate. After the information is entered, the causalBuilder prototype produces genuine causal statements that can be exported in different formats.

## Introduction

A molecular causal interaction describes the regulatory effect of a biological ‘source’ entity on the activity of a biological ‘target’. It represents a specific molecular event that has been interpreted or translated into a statement, providing details about the involved biomolecules and their interaction. Such ‘causal statements’ correspond to experimentally verifiable regulatory hypotheses that can form the basis for studying the behavior of highly complex biological networks. Causal statements can include diverse contextual information that detail the specifics of the activity states of the biomolecules involved and the biological background in which an interaction was observed and therefore can be assumed to be true (e.g. phosphorylations, taxon, tissue type, cell line, and experiment). The availability of these details provides data users with better criteria for deciding which causal interactions to include in their studies. For example, when computational modelers assemble the regulatory network of a specific cancer type, they can only determine which interactions are relevant when the metadata describing the exact biological context information supports that the causal statement is valid in the modeling context.

Recently, the Minimum Information about a Molecular Interaction Causal Statement (MI2CAST) has been developed to guide the curation of causal statements (1). The MI2CAST checklist serves as a framework for standardizing the production of high-quality causal interaction information, in several ways. First, it guides the biocurators who examine publications and extract knowledge about causal interactions to create high-quality, context-rich annotations (2). Second, it helps experimental biologists to consider a list of criteria related to experimental design and reporting, so that they can maximize the usefulness of their data. Third, it helps computational biologists, by declaring and formalizing the contextual information that is relevant for their models.

The process of biocuration is commonly performed with curation tools customized to capture the curation details associated with one specific database (3, 4). In principle, data generated with another curation interface can also be added to this database, provided that all mandatory annotation details are included by that curation interface in a format that can be mapped to this database. This has, for example, been realized by the PSI-MI (Proteomics Standards Initiative, Molecular Interaction (5)) with the development of the CausalTAB standard (also called PSI-MITAB2.8 (6)) for exchanging curated causal information.

This inspired us to move the MI2CAST checklist from theory to practice, by implementing the MI2CAST standard in a user-friendly and customizable curation tool: the causalBuilder prototype. This tool can be configured to cover both the essential metadata needed for a causal statement and any relevant subset of its many non-mandatory features. Furthermore, causalBuilder can export the full set of annotated details for a curated molecular interaction into a format that is compliant with several major data repositories. However, it should be noted that causalBuilder does not constitute a database to store these annotations.

In addition, causalBuilder functions as one of the first test-case applications that utilizes Visual Syntax Method (VSM (7)), a new curation technology that enables annotation of information of any type and of any complexity. VSM represents information in a form that is both intuitive for curators and semantically precise for computer processing. It uses an elementary conceptual model, combined with a general-purpose user interface, called ‘vsm-box’ (https://github.com/vsm/vsm-box (8), with lowercase ‘vsm’ as per JavaScript module naming convention). A vsm-box is a sophisticated input-component that can be embedded in a web page. As VSM allows the representation of knowledge in the form of a structured language, each vsm-box can hold a single unit of information: a VSM-sentence (with uppercase ‘VSM’ in reference to the VSM model). A vsm-box allows a curator to enter VSM-terms (interface elements that represent entities, relations, numbers, etc.) and connect them with VSM-connectors (specifying a syntax of how VSM-terms interrelate). Specifically, a VSM-term couples a human-friendly representation (readable text) with a unique identifier (stored in the background) taken from ontologies (e.g. Gene Ontology (9)), controlled vocabularies (e.g. PSI-MI (10)) and a collection of biological entities or relations (e.g. UniProt (11) or Relation Ontology (12)). To facilitate curation, a vsm-box may be pre-filled with a VSM-template: a combination of pre-generated VSM-terms, VSM-connectors and empty input-fields. Empty fields are VSM-term elements that still need to be filled in, and they provide autocomplete assistance that can be configured for term and identifier lookup from specific lists or server APIs (Application Programming Interfaces). Templates can resemble readable sentences in which curators only need to fill the empty fields, supported by semantic autocomplete lookup and by a visual, syntactic overview of how all annotation terms are connected together.

CausalBuilder’s main objective and scientific scope is to demonstrate the dynamic generation of curation templates that conform to the MI2CAST standard. The templates are based on the curation technology VSM and can easily be tailored to the particular needs of a causal-interaction curation task, as mandated by the available experimental details. The causalBuilder prototype presents the generated templates in a vsm-box interface where they can be evaluated by curation-tool developers or filled in by curators. Additionally, it already supports the export of the annotated data for individual causal statements into the VSM-JSON, causal-JSON or CausalTAB (PSI-MITAB2.8) formats.

## Implementation and use

**Figure 1.**
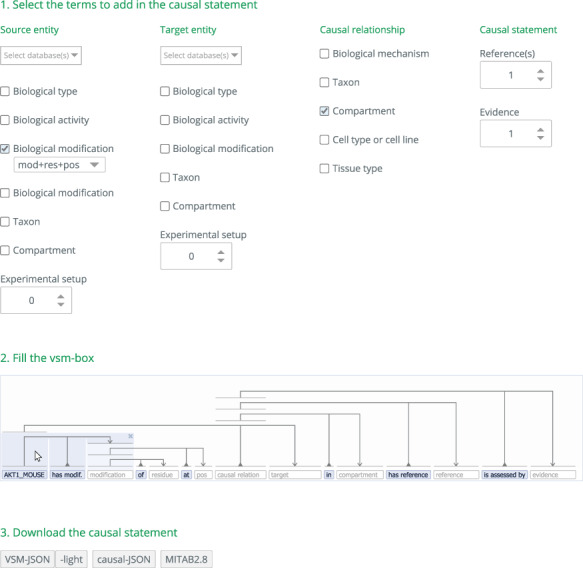
**Steps for building a causal statement in causalBuilder.** In Step 1, the different annotation features supported by MI2CAST can be selected in causalBuilder's interactive selection panel (larger than shown). Here, a ‘biological modification’ for the source entity is selected as well as the ‘compartment’ where the causal relationship occurs. In Step 2, the VSM-template that is created based on these selections contains the requested fields that need to be filled with annotations. The panel shows the mandatory field for ‘source entity’(already filled with an ‘AKT1_MOUSE’ annotation), and other empty fields for ‘[biological] modification’, ‘causal relation’, ‘target [entity]’, ‘compartment’, ‘reference [paper]’, and ‘evidence’ (hinted in lightgrey). Some VSM-terms are pre-filled (e.g., ‘has modif.’) and define the relation between empty fields. On top, pre-added VSM-connectors assign each term to one or more semantic subunits (all groups of three here); and note that connectors always attach to terms, not to other connectors. Mousing over the VSM-connectors highlights terms connected together in a triple. Here, it shows that the ‘modification’ term relates to the ‘AKT1_MOUSE’ source entity. As text is being typed as a VSM-term, an autocomplete panel appears. It presents matching terms and their description (a combination of definition, identifier, etc.), fetched from online controlled vocabularies relevant for that field, facilitated by the UniBioDicts software (see Step 2, below) (13). In Step 3, after the template is filled, the resulting causal statement can be downloaded in several formats.

CausalBuilder supports the MI2CAST checklist by enabling the on-demand creation of a data entry VSM-template to fully annotate and contextualize a molecular causal interaction, following the biological knowledge provided by a paper. As described in Figure 1, a causal statement is built in three steps: (i) selecting the annotation details to include in the causal statement, (ii) filling the VSM-template’s empty fields with appropriate annotation terms and (iii) downloading the annotated causal statement in various data formats.

### Step 1: Selection of the types of information to annotate

The MI2CAST guidelines define both mandatory and ‘optional’ rules. The mandatory unit of information to annotate during the manual curation of a causal interaction demands a source entity, a target entity and a causal relationship. In addition, at least one reference and an evidence type must be provided. These mandatory information fields are present by default in the template. The guidelines also specify several non-mandatory but nonetheless valuable contextual information types. These relate to the source or target entity (e.g. biological type, location, protein modifications and details about these modifications) or to the causal relationship (e.g. biological mechanism, cell line, and tissue type), but are contingent on the experimental details that are provided in a paper.

Depending on the information a user discovers while curating a paper, he/she can decide for each non-mandatory feature whether or not to include it for an annotation task, by selecting the corresponding checkbox or choice-list in causalBuilder’s configuration panel. Based on the selected features, this panel will dynamically generate additional choices for subfeatures when these become relevant (e.g. a phosphorylation’s position). Through this panel, the user determines the addition or removal of specific VSM-terms (filled or still empty) in the generated VSM-template placed in the vsm-box underneath. Hence, causalBuilder allows for a flexible selection of any amount of these non-mandatory terms, tailored to the annotation task.

In the generated template, the way that terms relate to each other is made clear by connectors (see Figure 1). For example, if ‘source’ and ‘target’ are each given their own ‘modification’ field, then each will be visually connected to the specific one that pertains to it. Likewise, if an entity has multiple ‘modification’ fields (e.g. for multiple phosphorylations) and these all have their own combination of ‘residue’ and/or ‘position’ subfeatures, then each subfeature will be linked to the correct ‘modification’ through a VSM-connector and a VSM-term, which makes the relation explicit and precise. This makes it easy for curators (and data users) to see what field belongs together with what other field and how.

### Step 2: Adding annotations to the VSM-template

When the types of information to annotate are selected, a user can fill in the resulting template with appropriate annotation terms. Each empty VSM-term contains a placeholder that hints what type of annotation should be filled in. Each empty field is also configured to perform term-lookup in appropriate biological vocabularies. This means that when a user starts typing the text in a field, an autocomplete panel appears that offers annotation options filtered down to term lists recommended by MI2CAST. For example, in the field for ‘causal relationship’, which expects the causal interaction’s regulation type, the autocomplete lookup is limited to terms from the PSI-MI controlled vocabulary (14) and Relation Ontology (12); so it could present, for example, ‘up-regulates (MI_2235)’, which is a term + identifier couple from PSI-MI. This enables the rapid selection of the desired vocabulary term by the curator and reduces the possible source of errors from typing or copy-pasting term text into forms.

This customized term-lookup is based on the vsm-box’s support for configuring each VSM-term. These customization settings are part of the generated template and can be inspected by double-clicking an empty term or mousing over a filled one. The term-lookup is also based on modules from the UniBioDicts open-source organization (https://github.com/UniBioDicts (13)). UniBioDicts provides unified access to a wide range of biological dictionaries (ontologies and controlled vocabularies), including those recommended in MI2CAST. These generically pluggable dictionaries can be easily linked to any input-field by generating a single line of template configuration code. Here, UniBioDicts makes these resources accessible through a single, virtual query-interface and translates all data on terms and identifiers returned by the various resources into a unified format, recognized by a vsm-box. For instance, the UBD Bioportal (https://github.com/UniBioDicts/vsm-dictionary-bioportal) calls BioPortal’s REST API web services (15) to access most of the ontologies recommended for annotations in MI2CAST.

### Step 3: Exporting the annotated causal statement

The VSM-template or VSM-sentence in the vsm-box can be downloaded at all times during the curation task, in VSM-JSON and VSM-JSON-light formats. The ‘light’ format is a subset of core data (including IDs and term names but excluding some template-related data like autocomplete filters) and constitutes a concisely formatted and human-readable essence of what the curator has annotated. Once the VSM-template is completely filled with the proper annotations, the curated causal statement can be downloaded as causal-JSON (https://github.com/vtoure/causal-json) as well, a format that can hold causal statements with all MI2CAST-supported annotation details. In addition, a standard PSI-MITAB2.8 (6) file can be generated, containing the subset of data supported by PSI-MI.

## Discussion

CausalBuilder advances the field of biocuration in several ways. First, it shows in practice how the concepts defined in MI2CAST to describe all aspects and context of molecular causal interactions, can be put into practice by biocurators through a clear and concise user interface. All information types described in MI2CAST are listed as annotation options and grouped into categories in the interface, which helps the curator to follow these guidelines easily and fully. As MI2CAST stipulates that certain annotation details depend on the depth of information available to the curator, this means that the curation template should be easily customizable to meet these available details. CausalBuilder fully supports this through the vsm-box software and by generating VSM-templates that are easily tailored to the specific annotation of particular combinations of MI2CAST-recommended information. The building of templates based on curation guidelines both facilitates and steers the curation process. Note that while causalBuilder has been implemented for the specific curation of causal interactions, one could nevertheless incorporate its interface into the existing curation platforms and extend it to enable the curation of non-causal information as well, with the same level of rigourous annotation, thanks to the flexibility offered by the VSM technology.

Second, we hope it serves as a demonstration for web developers who may become inspired to incorporate the causalBuilder interface (or vsm-box components in general) in their existing or future curation platform. For example, the causalBuilder prototype could be adapted with ‘one-click presets’ that configure the entire selection panel, to allow the generation of templates that cover the specific features and vocabularies required by their particular database. In that context, we would like to stress again that the causalBuilder prototype interface is not bundled with utilities that would manage, for example, quality control and archiving needs, as these would require the presence of a database backend and therefore the creation of an individual curation platform. When connected to an existing curation platform, compliance with completeness of the annotation requirements (both MI2CAST and platform-related) and inter-annotator agreement could be checked on the database side. Our prototype merely supports the export to several established formats including CausalTAB (PSI-MITAB2.8), which enables the transfer of single annotated causal statements to the existing databases, such as SIGNOR (16, 17) and IntAct (3). The causalBuilder prototype also supports export to causal-JSON, but future work could include export to MI-JSON (schema available at https://github.com/MICommunity/psi-jami/blob/master/ja-mi-interactionviewer-json/schema/mi-json-schema.json, 18 January 2021, date last accessed). This format also enables storage of causal interaction statements, although in its current state, it is not as flexible as causal-JSON. For instance, the use of ontologies is not as extensible in MI-JSON as in causal-JSON, and annotation of some terms is not yet supported. We are collaborating with the MI community to improve these gaps in order to provide users with a single JSON (standard) format enabling the support of causality that complies with MI2CAST.

Third, causalBuilder constitutes an early-adopter application of the VSM knowledge representation and curation technology and demonstrates a particular, template-based use. It is our hope that the causalBuilder example may inspire managers of curation projects to deploy the vsm-box in their curation platform, to allow their curators to benefit from the ease and flexibility of the VSM technology. The availability of the vsm-box resource (https://github.com/vsm/vsm-box (8)) facilitates the development of dedicated VSM-template annotation entry interfaces customized to the needs of various curation projects.

Furthermore, note that we programmed causalBuilder to dynamically insert or remove MI2CAST-specific annotation fields in the template, based on selection panel settings (e.g. multiple ‘evidence’ fields based on a counter), and also to insert extra subfeatures in the selection panel as needed (e.g. each new ‘modification’ needs a new selection list for subfeatures). As causalBuilder is the first project that implements such tailored VSM-template generation, it therefore also serves as an inspiration for how this process might be generalized in a fully automated design. As future work, a proper definition of a ‘meta-template’ (e.g. covering all possible MI2CAST template variants) could underpin the fully automated generation of both VSM-template graphs and selection panel features, subfeatures, etc., as these become relevant. A generalized template-generator could, for instance, support the template-based annotation of protein complexes with nested sub-complexes of proteins that may each have modifications.

## Conclusion

In summary, causalBuilder offers an intuitive, web-based prototype for the curation of a MI2CAST-compliant, molecular causal interaction and for the production of an annotated causal statement. The two major achievements of causalBuilder are to provide a MI2CAST-based testing ground for biocurators and software developers and to form a reference basis for the new applications and developments of VSM.

## Supplementary Material

baaa107_Supp

## Availability and implementation

The causalBuilder application is available at https://mi2cast.github.io/causalBuilder and the source code is available at https://github.com/mi2cast/causalBuilder under the Affero GPL license.
