# Supplementary material for: CausalBuilder: bringing the MI2CAST causal interaction annotation standard to the curator
Source: Database (Oxford). 2021 Feb 24;2021:baaa107. doi: 10.1093/database/baaa107 (PMC7904049; doi:10.1093/database/baaa107)

Supplementary File

The supplementary file showcases multiple examples of possible generated templates and annotations in causalBuilder. This aims to comprehend the wide range of annotation combinations that can be made with causalBuilder and more specifically, the VSM technology.

Supplementary Figure 1. This example describes the annotation of a causal statement where the biological activities (‘protein kinase A regulatory subunit binding activity’ and ‘protein kinase activity’) of both the source (3’,5’-cyclic AMP) and target (human KAPCA) entities have been specified.

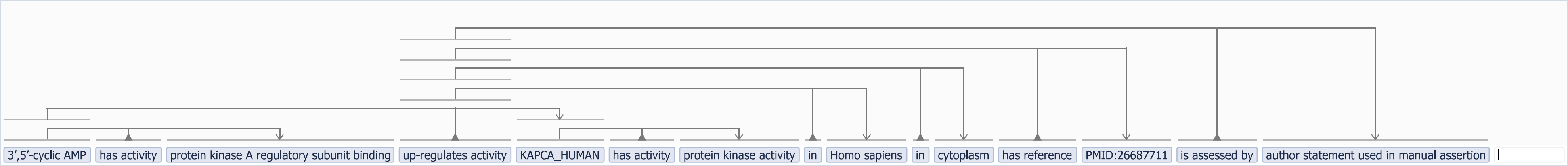

Supplementary Figure 2. This example shows the annotation of a causal statement where the target entity gets phosphorylated, shown by the ‘phosphorylation reaction’ mechanism. The causality occurs specifically in the breast cancer cell line.

Note that we also left away the surrounding vsm-box interface here, in order to create a figure that focuses on the curated VSM-sentence alone. For this, we copied the VSM-JSON generated by causalBuilder, pasted it at https://vsm.github.io/demo, and used it to generate a ‘white box’ styled SVG vector image.

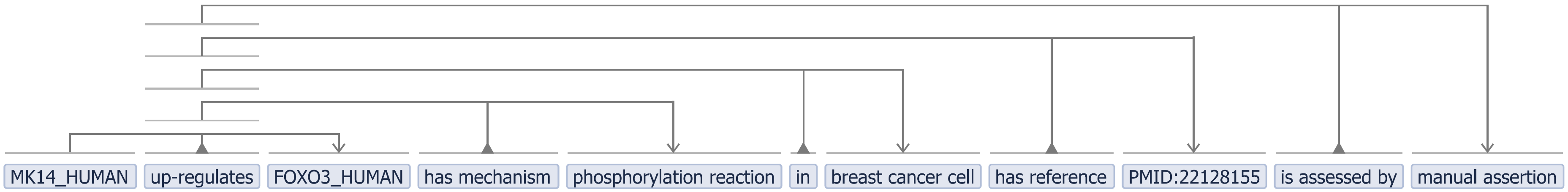

Supplementary Figure 3. This example shows a curation template produced by selecting a biological activity for the target entity (i.e., the target's specific activity that is affected by the causal interaction), in combination with two separate biological modifications for this target entity (e.g. to fill in a protein phosphorylated at two different sites, here both characterized by peptide position). A mouse cursor hover-highlights a chosen VSM-connector, to showcase that one can easily inspect which terms form a triple together, no matter how many other terms are in between them. The other empty fields are remaining annotation terms required by MI2CAST.

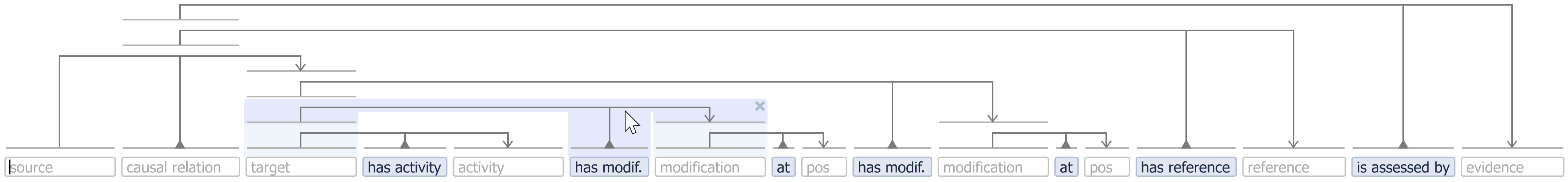

Supplement: baaa107_Supp [file baaa107_supp.zip › SupplementaryFiles.pdf]
